# 

**Published:** 1892-05-14

**Authors:** 


					PRICE TWOPENCE.
Vol. XII.?May 14th, 1892.
WITH EXTRA NURSING SUPPLEMENT.
Per Annum, 8s. 6d., post free.
Monthly Farts, 6d.; post free, 8d,
Ditto per Annun, 8s., post free.
O
IMICH HOSELTAA
WITH EXTRA NURSING SUPPLEMENT.
No. 29fr* Vol. XII.] Saturday,
May 14th, 1892.
[Twopence.

				

